# Effect of Heat Exposure on Activity Degradation of Enzymes in Mango Varieties Sindri, SB Chaunsa, and Tommy Atkins during Drying

**DOI:** 10.3390/molecules25225396

**Published:** 2020-11-18

**Authors:** Adnan Mukhtar, Sajid Latif, Joachim Mueller

**Affiliations:** 1 Institute of Agricultural Engineering (440e), Tropics and Subtropics Group, University of Hohenheim, Garbenstrasse 9, 70599 Stuttgart, Germany; sajid.latif@yahoo.com (S.L.); joachim.mueller@uni-hohenheim.de (J.M.); 2 Institute of Horticulture Sciences, University of Agriculture, Faisalabad, Sub-Campus Depalpur Okara, Renala Khurd 56300, Pakistan

**Keywords:** heat-sensitive enzyme, convective drying, Sindri, Samar Bahisht Chaunsa, Tommy Atkins

## Abstract

Mango has been described as a valuable source of nutrients and enzymes that are beneficial to human health. Drying at different temperatures not only affects the nutritional properties but can also contribute to the degradation of valuable enzymes in dried fruit. The novelty of this paper is to investigate the quality of hot air dried mango in terms of activity retention of the heat-sensitive enzymes (HSE). For this, HSE was first screened in fresh mango flesh of the variety Samar Bahisht (SB) Chaunsa. Later, the combined effect of different drying temperatures (40 °C, 50 °C, 60 °C, 70 °C, and 80 °C) and air velocities (1.0 ms^−1^ and 1.4 ms^−1^) on the activity retention of HSE in dried mango slices of the varieties Sindri, SB Chaunsa, and Tommy Atkins were investigated. The results showed that the drying temperature had a significant impact on the degradation of HSE, while at the same time some influence of the air velocity was also observed. Drying at 40 °C and an air velocity of 1.4 ms^−1^ retained more HSE compared to those samples dried at higher temperatures. The least retention of HSE was found in samples dried at 80 °C.

## 1. Introduction

Mango (*Mangifera indica* L.) is one of the most popular fleshy stone fruits. It is native to South Asia, from where it has been distributed worldwide to become the most cultivated fruit in the tropics [1]. With an annual production of over 54.6 million tons, mango ranked sixth among the major fruit crops [2]. In recent years, the world demand for mango has been increasing rapidly both for fresh and for processed fruits. The primary mango producing countries in the world are India, China, Thailand, Pakistan, Mexico, and Indonesia [3].

Mango flesh is of great importance due to its rich nutritional value as well as being a good source of health-promoting enzymes including antioxidant enzyme (catalase), also known as a perfect catalyst, and the digestive enzymes amylase and invertase [4,5]. Catalase is believed to be a key antioxidant enzyme and helps to protect cells from toxic reactive oxygen species. It is vital for good health to maintain an adequate level of the catalase enzyme and to replenish the depletion brought on by aging, strenuous exercise, and environmental conditions [6]. The amylase and invertase are necessary for the digestion process as they break down complex molecules into simpler forms. If the human body is unable to produce enough digestive enzymes, it can lead to digestive disorders [7]. Thus, incorporating foods into our diet with high natural enzymes like mango might be an important part of a dietary regimen to maintain health and wellness.

Mango, however, is highly perishable and deteriorates very fast after harvest due to ripening and putrescence. Therefore, numerous processing efforts have been made to offer an alternative to their fresh consumption. To prolong their availability on the market, hot air drying is one of the most common techniques used to reduce the moisture content to a level that prohibits the growth of microorganisms [8]. Mango and other tropical fruits are commonly dried to a target moisture content below a 15% wet basis at air temperatures between 40 and 80 °C and air velocity around 0.2–2.0 ms^−1^ and specific humidity of 10–25 g/kg [8,9,10,11,12,13,14,15]. Many experimental studies have proven that hot air drying of fruit is significantly influenced by the drying temperature and air velocity, whereas the specific humidity did not have a major impact on the drying rate [8,11,16,17]. Furthermore, many researchers have extensively documented the influence of these drying conditions on quality attributes such as color, texture, total soluble solids (TSS), starch, sugar, moisture content, fibers, phenolic contents, vitamins, and antioxidants of the dried mango [9,18,19,20,21,22,23,24]. In most of these studies, it was well observed that drying at temperatures of 70 °C and 80 °C contributed to rapid degradation of the fruit’s nutritional components, whereas this loss was greatly reduced at temperatures of 40 °C to 60 °C. Although the effects of different drying conditions on product quality are well studied, there is still a lack of information related to enzymatic activity retention, particularly in the varieties Sindri, Samar Bahisht Chaunsa, and Tommy Atkins. Since enzymes are mainly proteins in nature, the inter- and intra-molecular bonds of proteins may be disrupted under different drying conditions and their efficacy may decrease. Consequently, obtaining information about the drying operating parameters and its relationship to the enzymatic activity retention in dried mango is highly important because of the health benefits of the enzymes.

Nowadays, the use of enzyme supplements is also becoming increasingly popular. Dehydrated mango slices that preserve high levels of active enzymes may serve as an effective healthy snack. The greatest challenge in the modern food industry is not only to minimize the chemical degradation reactions but also to maximize the conservation of beneficial enzymes during drying. The objective of this study was to investigate and compare the most influential parameters of hot air drying, such as temperature and air velocity on the activity retention of heat-sensitive enzymes in dried mango slices. To screen the heat-sensitive enzymes, the initial selection was based on the enzymes that are most commonly studied in fresh mango of different varieties and are present in a concentration that is easy to measure. Therefore, the enzymes selected for this research work were catalase, polyphenol oxidase (PPO), amylase, and invertase [25,26,27,28]. After measuring the sensitivity level for each of the selected enzymes, we further evaluate the quality of hot air dried mango in terms of activity retention of heat-sensitive enzymes. The results from this research work will also provide a scientific basis to select the appropriate drying conditions to retain a maximum of heat-sensitive enzymes in dried mango.

## 2. Results and Discussion

### 2.1. Effect of Incubation Temperature on Enzyme Activity in Fresh Mango

**Figure 1:**
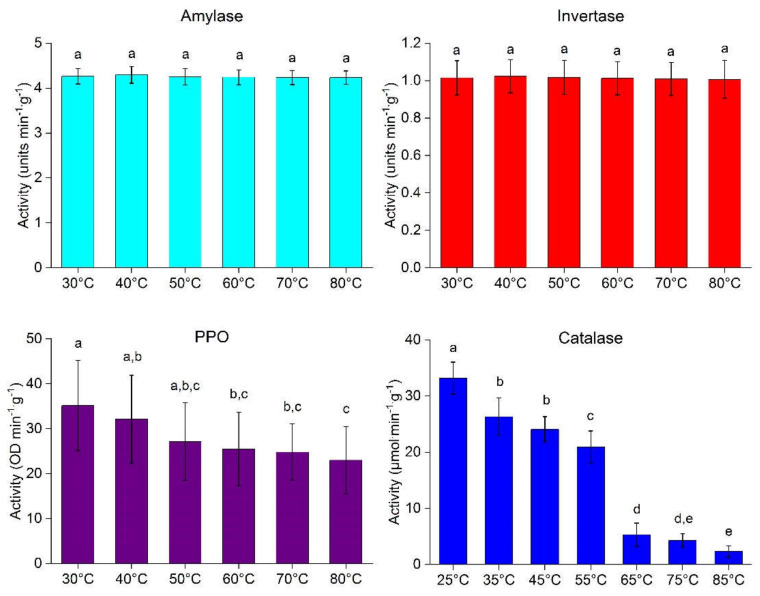
Enzyme activity of amylase, invertase, polyphenol oxidase (PPO) and catalase of mango variety Samar Bahisht (SB) Chaunsa after 10 min incubation of enzyme extract at various temperatures. ^a–e^ values with different letters in the graph represent significant differences *p* < 0.05, (*n* = 18 × 3). Multiple comparisons Tukey honestly significant difference (HSD) test reports for PPO and catalase are provided in Appendix A Table A1 and Table A2, respectively.

Figure 1 presents the enzymatic activity of amylase, invertase, polyphenol oxidase (PPO), and catalase obtained after incubating extracted mango samples for 10 min at different temperatures.

Amylase and invertase revealed a high heat tolerance, as there was no significant difference (*p* > 0.05) in activity measured at different temperatures. The highest amylase activity (4.29 units min^−1^·g^−1^) and invertase activity (1.02 units min^−1^·g^−1^) were observed at 40 °C. However, no significant differences could be found between 30 °C and 80 °C. Similar information on the thermostability of amylase activity in mango peel by Mehrnoush et al. [29] and invertase from *Aspergillus caespitosus* under culture conditions of submerged or solid-state fermentation was also reported [30].

While amylase and invertase showed no effect of incubation temperature, the activity of PPO and catalase was considerably reduced by increased temperature. The PPO activity of 35.20 OD min^−1^·g^−1^ at 30 °C decreased to 23.00 OD min^−1^·g^−1^ at 80 °C, representing a 34.66% reduction. These findings are in agreement with the results reported by different scientists on heat inactivation of PPO enzyme in different fruits and vegetables [31,32,33]. The catalase activity at 25 °C of 33.20 µmol·min^−1^·g^−1^ gradually decreased to 2.33 µmol·min^−1^·g^−1^ at 85 °C, representing a 92.98% reduction in catalase activity. Wang [34] found a similar effect of temperature on the catalase activity in chilled zucchini squash. Based on the screening results, the catalase was found as a more heat-sensitive enzyme and selected as our target enzyme. However, PPO also behaves somehow like catalase and the information about the PPO enzyme is important during storage of dried products. Hence, PPO and catalase as heat-sensitive enzymes were chosen to proceed with the drying experiments.

### 2.2. Polyphenoloxidase (PPO) Activity of Fresh and Dried Mango

**Figure 2:**
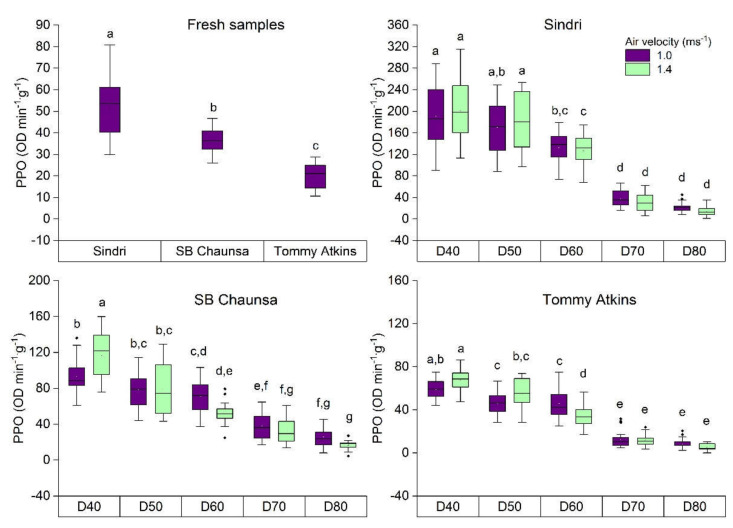
Polyphenol oxidase (PPO) activity in fresh and dried mango of the varieties Sindri, SB Chuansa, and Tommy Atkins; D = dried samples at temperatures 40 °C to 80 °C. ^a–g^ values with different letters in the box plot indicate the significant difference *p* < 0.05, (*n* = 20 × 3).

PPO activity was measured in fresh and dried samples of varieties Sindri, Samar Bahisht (SB) Chaunsa, and Tommy Atkins (Figure 2).

The maximum PPO activity was measured in fresh samples of Sindri (30.00–80.89 OD min^−1^·g^−1^) followed by SB Chaunsa (26.00–46.67 OD min^−1^·g^−1^) and Tommy Atkins (10.67–28.87 OD min^−1^·g^−1^). However, in the dried samples of all three varieties, low drying temperature 40 °C and high air velocity 1.4 ms^−1^ retained the maximum PPO activity of Sindri (113.37–315.20 OD min^−1^·g^−1^), SB Chaunsa (75.97–159.85 OD min^−1^·g^−1^), and Tommy Atkins (TA) (47.60–86.17 OD min^−1^·g^−1^) compared to mango dried at 50 °C to 80 °C. The results obtained from this study correspond with those found in fresh mango [5,26] and the heat inactivation of PPO enzymes in different fruits [32,35,36]. min^−1^·g^−1^.

PPO is an important enzyme that has numerous health-promoting properties including antiviral, anticarcinogen, antimicrobial, and antimutagenic agents [37,38,39] and can be used in several industrial applications [40,41]. However, this enzyme is also known to be responsible for the enzymatic browning of fruits [42]. Drying up to a safe water activity level of a_w_ ≤ 0.6 is required to inactivate this enzyme [32]. Moreover, it is also important to understand that mango slices dried at 40 °C not only preserve the beneficial enzymes but also those enzymes that are involved in the oxidation of the phenolic compounds or are susceptible to color deterioration. Therefore, the mango slices dried at 40 °C should be packaged and stored appropriately.

### 2.3. Catalase Activity of Fresh and Dried Mango

**Figure 3:**
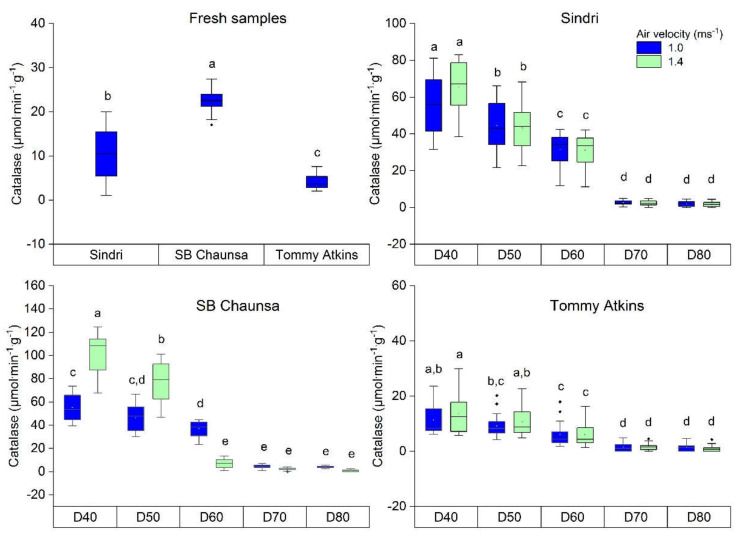
Catalase activity in fresh and dried mango of the varieties Sindri, SB Chuansa, and Tommy Atkins, respectively; D = dried samples treated at temperatures 40 °C to 80 °C. ^a–e^ values in box plot with different superscripts are significantly different under the limit of *p* < 0.05, (*n* = 20 × 3).

Figure 3 shows the catalase activity of fresh and dried mango of the varieties Sindri, SB Chaunsa, and Tommy Atkins. When comparing the fresh samples of all three varieties, the maximum catalase activity was observed in SB Chaunsa (17.03 to 27.42 µmol·min^−1^·g^−1^) followed by Sindri (8.73 to 19.5 µmol·min^−1^·g^−1^) and Tommy Atkins (2.08 to 7.58 µmol·min^−1^·g^−1^).

The varieties Sindri and SB Chanusa had a higher catalase activity in the fresh samples compared to Tommy Atkins, which might be due to varietal differences, different climatic origins, compositional variations, and fruit maturity stages. These results are also corresponding to research on changes in catalase activity during storage and ripening of the fruits [43,44]. Since catalase activity is closely associated with the ripening and maturity of fruit, the difference in catalase activity of the three varieties can be related to the brix values as presented in Table 1. However, after drying, the quality of the final dried mango was evaluated in terms of residual catalase activity as shown in Figure 3. As expected, at different drying temperatures and air velocity of 1.0 ms^−1^, the highest level of catalase activity in Sindri (31.63–81.17 µmol·min^−1^·g^−1^), SB Chaunsa (39.29–73.67 µmol·min^−1^·g^−1^), and TA (6.22–23.62 µmol·min^−1^·g^−1^) was retained in the product dried at 40 °C. However, when using higher drying temperatures of 50 °C, 60 °C, 70 °C, and 80 °C, the activity retention of catalase decreased significantly (*p* < 0.05). Particularly, at drying temperatures of 70 °C and 80 °C, most of the catalase activity was lost, presumably by denaturation of the enzyme. The least retention of catalase (0.00–5.71 µmol·min^−1^·g^−1^) was noticed in samples dried at 80 °C. Vega-Gálvez et al. [17] reported that during drying of fruits, the cellular damage is less at a lower temperature while at higher temperatures, thermal destruction predominates and weakens the cell tissues. Therefore, drying at high temperatures causes more destruction of heat-sensitive enzymes.

The experimental results also highlighted that the tendency to activity retention of catalase in dried mango changed as the drying-air velocity was raised to 1.4 ms^−1^. Drying at a lower temperature of 40 °C and an air velocity of 1.4 ms^−1^ can save 10.64% to 40.62% more enzyme units compared to the samples dried at an air velocity of 1.0 ms^−1^. A significant difference was noticed in SB Chaunsa while in Sindri and Tommy Atkins the enzyme units were insignificantly higher. This might be attributed to the fact that a higher air velocity decreases the drying time at the same drying temperature (Table 1). Similar observations about influence of air velocity in decreasing drying time were also reported by Putra and Ajiwiguna [8]. The activity of heat-sensitive enzymes is also directly related to heat treatment time [45]. As a result, drying at a low temperature of 40 °C and an air velocity of 1.4 ms^−1^ retained more enzyme units. Thus, to preserve the maximum activity of the heat-sensitive enzymes in dried mango, the drying temperature and air velocity are both important parameters to be considered.

Many researchers have also reported that drying fruits at low temperatures could have a beneficial impact on the preservation of the structure [46], flavor [14], protein [47], antioxidants [48], ascorbic acid [49,50], and total phenolic compounds [22,51]. Catalase is believed to be a key antioxidant enzyme in the human body’s defense against oxidative stress [52]. Preservation of this enzyme in dried mango is of great importance because of its strong antioxidant properties [6]. By preserving the heat-sensitive enzyme (catalase) in dried fruit, it might also be possible to preserve other nutritional components described. Instead of measuring a variety of quality parameters, catalase activity in dried mango could be used as an indicator of quality deterioration due to excessive heat exposure during drying. It has also been documented in a previous study that hot air drying is economically more viable compared to other drying methods like freeze-drying [53]. Therefore, the use of hot air drying to preserve active enzyme units in dehydrated mango might provide an extra advantage to minimize its drying costs.

### 2.4. Drying Kinetics of Catalase Activity

**Figure 4:**
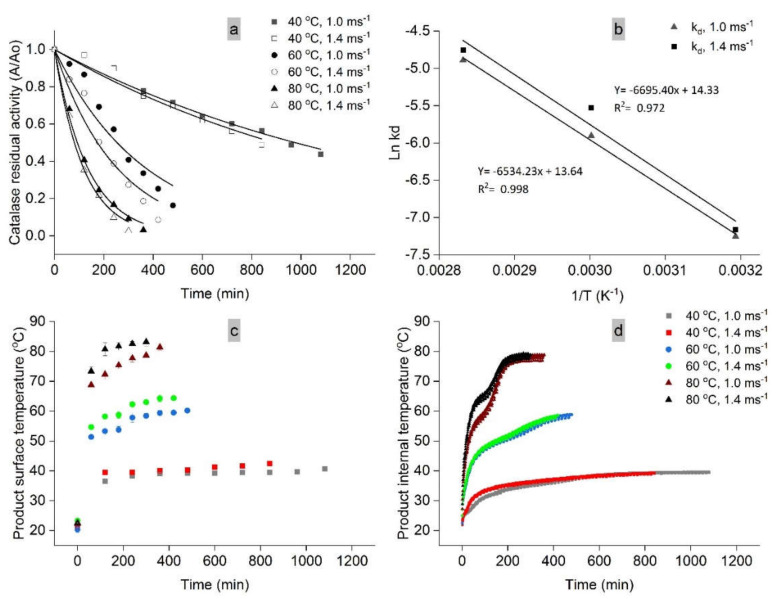
(**a**) Kinetics of catalase activity; (**b**) Arrhenius plot for the energy of deactivation of catalase; (**c**) product surface temperature; (**d**) product internal temperature during drying of mango variety Sindri at 40 °C, 60 °C, and 80 °C; air velocity 1.0 ms^−1^ or 1.4 ms^−1^.

For a better understanding of the catalase degradation rate, the residual catalase activity during mango drying was evaluated in the variety Sindri. The results are displayed in Figure 4a,b.

At a constant air velocity of 1.0 ms^−1^, the drying temperatures 40 °C, 60 °C, and 80 °C exhibited a destruction rate k_d_ of 0.00071, 0.00273, and 0.00751 min^−1^, respectively. The degradation rate accelerated 3.84-fold during drying at 60 °C and 10.58-fold at 80 °C, compared to 40 °C. Similar explanations regarding enzyme inactivation at different temperatures were also found in previous studies [33,54,55]. In addition, when drying at 40 °C, 60 °C, and 80 °C, an increase in air velocity of 1.4 ms^−1^ increased the destruction rate k_d_ by 8.77–31.42%. These results can be well explained by Figure 4c,d. At an air velocity of 1.4 ms^−1^, the product reached its target temperature faster, and higher surface and internal temperatures were observed compared to an air velocity of 1.0 ms^−1^. Vega-Gálvez et al. [17] also reported that the heat diffusion capacity of a high air velocity was more pronounced than that for a low one and therefore drying at an air velocity of 1.4 ms^−1^ causes a higher destruction of enzymes. These findings were further clarified by comparing the Arrhenius plot of deactivation energy (E_d_) for both, 1.0 ms^−1^ and 1.4 ms^−1^ air velocity (Figure 4b). Air velocity of 1.4 ms^−1^ exhibited a 2.41% higher E_d_ value of catalase compared to a 1.0 ms^−1^ air velocity, which confirms a higher enzyme destruction at high air velocities. However, these findings are in contradiction to our results as explained in Figure 3, where the maximum catalase activity was retained in mango samples dried at 40 °C and an air velocity of 1.4 ms^−1^. This is because of the fact that the activity of heat-sensitive enzyme (catalase) is also directly related to the heat treatment time. Drying at 40 °C and an air velocity of 1.4 ms^−1^ required less time (120–195 min) to dry up to the target moisture content compared to a 1.0 ms^−1^ air velocity (Table 1). Thus, drying at a low temperature of 40 °C and an air velocity of 1.4 ms^−1^ was successful in saving more enzyme units, although the destruction rate was comparatively higher than the air velocity 1.0 ms^−1^. Consequently, catalase activity is directly affected by the drying air temperature, velocity, and heat treatment time. Therefore, its activity can be used as a control parameter to predict changes and improve the drying operations.

## 3. Materials and Methods

### 3.1. Mango Varieties

Fresh, medium ripe mango (*Mangifera indica* L.) in export quality of the varieties Sindri and Samar Bahisht (SB) Chaunsa were harvested in Khanewal, Pakistan (30°20′13.9″ N, 71°54′35.0″ E) and transported by air to Germany. In addition, fruits of the variety Tommy Atkins were obtained from a local market in Stuttgart, Germany. During experiments, the mangoes were stored under refrigeration at a temperature of 11 ± 1 °C for not more than six weeks.

### 3.2. Enzyme Activity Assays

#### 3.2.1. Catalase

The catalase activity was determined by the megazyme assay kit (K-CATAL, Romer Labs GmbH, Butzbach, Germany). A decrease in H_2_O_2_ concentration was observed following an incubation of the extracted mango samples with a H_2_O_2_ standard solution. The reaction was stopped by adding 15 mmol sodium azide, which strongly inhibits catalase. The remaining concentration of H_2_O_2_ was measured using an enzyme-linked colorimetric detection method employing 3,5-dichloro-2-hydroxy-benzenesulfonic acid (DHBS), 4-aminoantipyrine (AAP), and peroxidase. The resulting quinoneimine dye was measured at 520 nm by a spectrophotometer (DR-6000, Hach Lange GmbH, Berlin, Germany).

$$2H_{2}O_{2}\overset{\mathrm{Catalase}}{\to }2H_{2}O\;+\;O_{2};$$

$$2H_{2}O_{2}\;+\;\mathrm{DHBS}\;+\;\mathrm{AAP}\overset{\mathrm{Peroxidase}}{\to }\mathrm{quinoneimine}\;\mathrm{dye}\;+\;4H_{2}O.$$

One unit of catalase activity is defined as the amount of enzymes required to decompose 1 μmol of H_2_O_2_ per min per gram of sample at 25 °C.

#### 3.2.2. Polyphenol Oxidase (PPO)

PPO activity was measured as described by Liu et al. [56]. In this method, a catechol solution (0.175 M) was used as a substrate because catechol oxidizes PPO is located in mango skin, sap, and pulp [57]. For the PPO activity assay, 2.5 mL of a freshly prepared catechol solution was mixed with 500 µL of the enzyme extract and incubated at 30 °C for 3 min. Then it was immediately transferred into a cuvette and the change in absorbance was measured at 495 nm by using a spectrophotometer (DR-6000, Hach Lange GmbH, Berlin, Germany). One unit of PPO activity was calculated by an increase in the absorbance of 0.001 per min per gram of sample at 30 °C.

#### 3.2.3. Amylase

Amylase activity was assayed according to the method described by Zakir et al. [58]. One percent (*w/v*) starch solution was used as a substrate. For the amylase activity assay, 2.9 mL starch solution was mixed with 100 µL of enzyme extract and the mixture was incubated at 37 °C for 10 min. The reaction was stopped by adding 1 mL of 3,5-dinitrosalicylic acid. Subsequent heating in boiling water for 5 min completed the reaction. The reaction mixture was allowed to cool for 15 min at room temperature and 9 mL of water was added to make a dilution. The mixture absorbance was measured at 540 nm by a spectrophotometer (DR-6000, Hach Lange GmbH, Berlin, Germany). The enzyme activity was measured by estimating the release of maltose calculated from the standard curve prepared by using maltose at concentrations of (0–6 mg·mL^−1^). One unit of enzyme activity was defined as the amount of enzymes required to release 1 mg of maltose per min per gram of sample at 37 °C.

#### 3.2.4. Invertase

Invertase activity was evaluated by estimating the release of glucose calculated from the standard curve prepared with different concentrations of glucose [58]. One percent (*w/v*) sucrose solution was used as a substrate. For the invertase activity assay, 2.9 mL of sucrose solution and 100 µl of enzyme extract were mixed and incubated at 37 °C for 10 min. The reaction was terminated by adding 1 mL of 3,5-dinitrosalicylic acid followed by heating in boiling water for 5 min to complete the reaction. After cooling at room temperature for 15 min, the mixture was diluted by adding 9 mL of water. The absorbance was recorded at 575 nm by a spectrophotometer (DR-6000, Hach Lange GmbH, Berlin, Germany) and the enzyme activity was calculated by comparing the standard curve prepared by using glucose at concentrations of (0–2.5 mg·mL^−1^). One unit of enzyme activity was defined as the amount of enzymes required to release 1 mg of glucose per min per gram of sample at 37 °C.

### 3.3. Incubation Trials to Determine the Heat-Sensitivity of Enzymes in Fresh Mango

The heat-sensitivity of the enzymes catalase, polyphenol oxidase (PPO), amylase, and invertase was investigated in fresh mango of the variety SB Chaunsa. The enzymes were extracted according to the modified method of Ndiaye et al. [36]. Eighteen mangoes were randomly selected and divided equally into three batches. Six extractions were prepared from each batch by mixing the flesh of six different mangoes. The fruits were cut into small pieces and homogenized with a vortex mixer (Polytron 2500E, Kinematica, Luzern, Switzerland). Next, 10 g (~1.8 g dry matter) of homogenate was transferred into each falcon tube adding 10 mL of extraction buffer (0.1 M Potassium phosphate buffer, pH 7.0). After mixing, the samples were further centrifuged (Z326K, HERMLE Labortechnik GmbH, Wehingen, Germany) at 13,400 rpm for 10 min at 4 °C. The supernatants were collected and run separately in triplicates to test the activity of enzymes after 10 min incubation at temperatures from 25 °C to 85 °C for catalase and 30 °C to 80 °C for PPO, amylase, and invertase. The temperature selection for the incubation trials was based on the optimal temperature range found in the literature. For instance, this optimum temperature range is stated to be between 25 and 50 °C for catalase [59], 30 and 50 °C for PPO [60], 40 and 70 °C for amylase [61], and 40 and 60 °C for invertase [62,63].

### 3.4. Hot Air Convective Thin-Layer Drying of Mango Slabs

The hot air drying experiments were carried out by using a laboratory convection dryer designed at the Institute of Agricultural Engineering, University of Hohenheim, Stuttgart, Germany [64], which allows a highly accurate control of the drying air temperature, humidity, and velocity. Prior to drying experiments, the average initial moisture content, degree brix, and water activity were analyzed in fresh mangoes (Table 1). The moisture content was determined by an oven method at 105 °C overnight [65], degree brix was measured by digital refractometer (ATAGO PR-201 palette, ATAGO Co. Ltd., Tokyo, Japan), and water activity using a hygrometer (Rotronic A2, Rotronic AG, Basserdorf, Switzerland) at 23 °C after 30 min of stability.

For drying, the mangoes were sliced in 4 cm × 2 cm × 0.8 cm pieces using a stainless-steel knife and a food dicer (MultiSchneider Serano 7, Ritter, Groebenzell, Germany). All the experiments were done in duplicates by using the overflow chamber of the dryer at 40 °C, 50 °C, 60 °C, 70 °C, and 80 °C, maintaining a constant specific humidity of 10 g kg^−1^ at air velocities of 1.0 ms^−1^ and 1.4 ms^−1^. The system was warmed up for 60 min to achieve constant drying conditions for the desired set points. An infrared thermometer (IR 260-8S, Voltcraft, Hirschau, Germany) was used to measure the fruit surface temperature at three different locations, while two thermocouple sensors (Mantel-Thermoelemente GTF 101, GHM Messtechnik GmbH, Regenstauf, Germany) of 0.5 mm in diameter were inserted to the center of the fruit slabs to record the inner temperature of the fruit during drying. The samples with initial mass of 330 ± 30 g were dried and weighed automatically at regular intervals of 15 min until the target moisture content of approximately 11% wet basis was achieved. This moisture content is equivalent to the hygienically safe water activity a_w_ ≤ 0.6. The time required for each of the drying experiments are also presented in Table 1. The dried mango slices were cooled, packed airtight in polyethylene bags, and stored in a dark place at room temperature for further analysis for a maximum period of two months.

### 3.5. Measurement of Enzyme Activity in Fresh and Dried Mango

Activity of heat-sensitive enzymes, identified in Section 3.3, was measured in fresh and dried samples of the varieties Sindri, SB Chaunsa, and Tommy Atkins, taking 120 mangoes per variety. For all three varieties, twenty extractions were prepared for each of the fresh and dried samples. For the preparation of each extraction from fresh samples, the flesh from six different mangoes was randomly selected and the supernatant was collected by following the same procedure as described in Section 3.3. However, for the dried samples, dried slices from the same treatment were randomly selected, cut into small pieces, and mixed well with the help of a homogenizer. Afterwards, 1.96 g (~1.8 g dry matter) of dried samples were mixed with 10 mL of extraction buffer and centrifuged under the previously described conditions (Section 3.3). The supernatant was collected and run separately in triplicates to test the enzymatic activity.

### 3.6. Modeling of Enzyme Activity Kinetics during Drying

Kinetic modeling describes the residual enzyme activity as a function of drying time and can be used to predict changes in product quality during drying. To measure the residual enzyme activity during drying, the samples were collected at regular 2-h-intervals at 40 °C and for 1-h-intervals at 60 °C and 80 °C. The enzyme degradations were expressed by a first-order kinetic model [66,67]:

A/A_o_ = exp (−k_d_ × t), (1)

where A is the residual activity after the treatment time t (min), A_o_ is the initial activity, and k_d_ is the deactivation or destruction rate constant (min^−1^). The deactivation rate constant (k_d_) is temperature dependent according to the Arrhenius equation [54]:

k_d_ = k_do_ × exp (−E_d_/RT), (2)

where k_d_ is the deactivation rate constant at the process temperature (min^−1^), k_do_ is the pre exponential factor, E_d_ is the energy of deactivations (J·mol^−1^), R is the universal gas constant 8.314 (J·mol^−1^·K^−1^), and T is the absolute temperature in (K).

### 3.7. Statistical Analysis

The statistical analysis was done by using the IBM statistical package SPSS 22.0. Mean differences in the treatments were tested for significance level (α = 0.05) using the ANOVA and Tukey’s honestly significant difference (HSD) method. The data were plotted by using Origin Pro 2020 (OriginLab Co., Northampton, MA, USA).

## 4. Conclusions

The combined effect of different drying temperatures and air velocities on the activity retention of the heat-sensitive enzymes catalase and polyphenol oxidase (PPO) in dried mango was observed. Results proved that the drying temperature had the greatest influence on the catalase degradation rate with some impact of the air velocity. Drying should be performed at a low temperature and a high air velocity in order to preserve the maximum enzyme units in dried mango. At the same time, however, drying at a lower temperature also preserved the maximum PPO units susceptible to color deterioration. Therefore, the dried mango should be stored at a proper place after adequate packaging. Alternatively, drying at higher temperatures with a high air velocity caused a drastic reduction of enzyme activity. These results highlight new possibilities to use catalase activity to compare drying temperatures and to optimize the drying technique. Moreover, the residual enzyme units in dried samples can be used as a new parameter to evaluate the quality of dried mango. Lately, industries have shown increased interest to find an optimum drying temperature to produce dried fruit with a good stability and high enzyme content. This study suggests the suitable temperature and air velocity to reduce enzyme destruction in dried mango products. Further research should be performed on the effect of storage time and packaging materials on enzyme stability. In addition, the correlation of enzyme activity with changes in heat-sensitive nutrients of mango during convective drying and the influence of maturity on enzyme activity needs to be investigated.

## Figures and Tables

**Table 1:** Brix, moisture content (MC) percentage wet basis, water activity (a_w_), and drying duration of mango varieties Sindri, SB Chaunsa, and Tommy Atkins at temperatures of 40 °C to 80 °C; air velocities 1.0 ms^−1^ and 1.4 ms^−1^.

Mango Varieties Fresh Samples				Drying Duration (Min)									
	Brix	MC	a_w_	40 °C		50 °C		60 °C		70 °C		80 °C	
		**(% wb)**		1.0 ms^−1^	1.4 ms^−1^	1.0 ms^−1^	1.4 ms^−1^	1.0 ms^−1^	1.4 ms^−1^	1.0 ms^−1^	1.4 ms^−1^	1.0 ms^−1^	1.4 ms^−1^
Sindri	17.33 ^b^ ± 1.71	86.96 ^b^ ± 2.05	0.922 ^b^ ± 0.012	915 ± 28.99	750 ± 26.87	660 ± 23.33	540 ± 20.51	465 ± 17.68	410 ± 16.97	350 ± 15.56	300 ± 12.73	280 ± 10.61	250 ± 9.90
SB Chaunsa	20.69 ^a^ ± 1.46	82.12 ^c^ ± 2.62	0.920 ^b^ ± 0.010	1155 ± 36.77	960 ± 31.11	750 ± 28.28	615 ± 25.46	540 ± 22.63	465 ± 21.21	390 ± 18.38	330 ± 16.26	310 ± 13.44	280 ± 11.31
Tommy Atkins	13.95 ^c^ ± 2.81	89.08 ^a^ ± 2.22	0.937 ^a^ ± 0.012	825 ± 26.16	705 ± 24.04	570 ± 21.92	465 ± 19.80	410 ± 14.14	360 ± 14.85	310 ± 12.73	270 ± 12.02	240 ± 7.78	210 ± 8.49

Data of fresh samples are presented as mean (*n* = 20 × 3) ± standard deviation; ^a–c^ values with different letters in a column indicate significantly different (Tukey’s HSD test, *p* < 0.05). Drying duration values are provided in duplication.

## Appendix A

**Table A1:** Multiple comparisons of polyphenol oxidase (PPO) activity at different temperatures.

Temperature		Mean Difference	Std. Error	Sig.	95% Confidence Interval	
					Lower Bound	Upper Bound
30 °C	40 °C	3.0372	2.83829	0.892	−5.2069	11.2814
	50 °C	8.0367	2.83829	0.060	−0.2075	16.2808
	60 °C	9.6667 *	2.83829	0.012	1.4225	17.9108
	70 °C	10.3822 *	2.83829	0.005	2.1381	18.6264
	80 °C	12.1972 *	2.83829	0.001	3.9531	20.4414
40 °C	30 °C	−3.0372	2.83829	0.892	−11.2814	5.2069
	50 °C	4.9994	2.83829	0.495	−3.2447	13.2436
	60 °C	6.6294	2.83829	0.190	−1.6147	14.8736
	70 °C	7.3450	2.83829	0.110	−0.8991	15.5891
	80 °C	9.1600 *	2.83829	0.020	0.9159	17.4041
50 °C	30 °C	−8.0367	2.83829	0.060	−16.2808	0.2075
	40 °C	−4.9994	2.83829	0.495	−13.2436	3.2447
	60 °C	1.6300	2.83829	0.992	−6.6141	9.8741
	70 °C	2.3456	2.83829	0.962	−5.8986	10.5897
	80 °C	4.1606	2.83829	0.687	−4.0836	12.4047
60 °C	30 °C	−9.6667 *	2.83829	0.012	−17.9108	−1.4225
	40 °C	−6.6294	2.83829	0.190	−14.8736	1.6147
	50 °C	−1.6300	2.83829	0.992	−9.8741	6.6141
	70 °C	0.7156	2.83829	1.000	−7.5286	8.9597
	80 °C	2.5306	2.83829	0.948	−5.7136	10.7747
70 °C	30 °C	−10.3822 *	2.83829	0.005	−18.6264	−2.1381
	40 °C	−7.3450	2.83829	0.110	−15.5891	0.8991
	50 °C	−2.3456	2.83829	0.962	−10.5897	5.8986
	60 °C	−0.7156	2.83829	1.000	−8.9597	7.5286
	80 °C	1.8150	2.83829	0.988	−6.4291	10.0591
80 °C	30 °C	−12.1972 *	2.83829	0.001	−20.4414	−3.9531
	40 °C	−9.1600 *	2.83829	0.020	−17.4041	−0.9159
	50 °C	−4.1606	2.83829	0.687	−12.4047	4.0836
	60 °C	−2.5306	2.83829	0.948	−10.7747	5.7136
	70 °C	−1.8150	2.83829	0.988	−10.0591	6.4291

Significance level *, indicate the *p*-values < 0.05 (Tukey HSD test).

**Table A2:** Multiple comparisons of catalase activity at different temperatures.

Temperature		Mean Difference	Std. Error	Sig.	95% Confidence Interval	
					Lower Bound	Upper Bound
25 °C	35 °C	6.8794 *	0.78998	0.000	4.5100	9.2489
	45 °C	9.1500 *	0.78998	0.000	6.7805	11.5195
	55 °C	12.2600 *	0.78998	0.000	9.8905	14.6295
	65 °C	27.8828 *	0.78998	0.000	25.5133	30.2523
	75 °C	28.9306 *	0.78998	0.000	26.5611	31.3000
	85 °C	30.8728 *	0.78998	0.000	28.5033	33.2423
35 °C	25 °C	−6.8794 *	0.78998	0.000	−9.2489	−4.5100
	45 °C	2.2706	0.78998	0.070	−0.0989	4.6400
	55 °C	5.3806 *	0.78998	0.000	3.0111	7.7500
	65 °C	21.0033 *	0.78998	0.000	18.6339	23.3728
	75 °C	22.0511 *	0.78998	0.000	19.6816	24.4206
	85 °C	23.9933 *	0.78998	0.000	21.6239	26.3628
45 °C	25 °C	−9.1500 *	0.78998	0.000	−11.5195	−6.7805
	35 °C	−2.2706	0.78998	0.070	−4.6400	0.0989
	55 °C	3.1100 *	0.78998	0.003	0.7405	5.4795
	65 °C	18.7328 *	0.78998	0.000	16.3633	21.1023
	75 °C	19.7806 *	0.78998	0.000	17.4111	22.1500
	85 °C	21.7228 *	0.78998	0.000	19.3533	24.0923
55 °C	25 °C	−12.2600 *	0.78998	0.000	−14.6295	−9.8905
	35 °C	−5.3806 *	0.78998	0.000	−7.7500	−3.0111
	45 °C	−3.1100 *	0.78998	0.003	−5.4795	−0.7405
	65 °C	15.6228 *	0.78998	0.000	13.2533	17.9923
	75 °C	16.6706 *	0.78998	0.000	14.3011	19.0400
	85 °C	18.6128 *	0.78998	0.000	16.2433	20.9823
65 °C	25 °C	−27.8828 *	0.78998	0.000	−30.2523	−25.5133
	35 °C	−21.0033 *	0.78998	0.000	−23.3728	−18.6339
	45 °C	−18.7328 *	0.78998	0.000	−21.1023	−16.3633
	55 °C	−15.6228 *	0.78998	0.000	−17.9923	−13.2533
	75 °C	1.0478	0.78998	0.838	−1.3217	3.4173
	85 °C	2.9900 *	0.78998	0.004	0.6205	5.3595
75 °C	25 °C	−28.9306 *	0.78998	0.000	−31.3000	−26.5611
	35 °C	−22.0511 *	0.78998	0.000	−24.4206	−19.6816
	45 °C	−19.7806 *	0.78998	0.000	−22.1500	−17.4111
	55 °C	−16.6706 *	0.78998	0.000	−19.0400	−14.3011
	65 °C	−1.0478	0.78998	0.838	−3.4173	1.3217
	85 °C	1.9422	0.78998	0.184	−0.4273	4.3117
85 °C	25 °C	−30.8728 *	0.78998	0.000	−33.2423	−28.5033
	35 °C	−23.9933 *	0.78998	0.000	−26.3628	−21.6239
	45 °C	−21.7228 *	0.78998	0.000	−24.0923	−19.3533
	55 °C	−18.6128 *	0.78998	0.000	−20.9823	−16.2433
	65 °C	−2.9900 *	0.78998	0.004	−5.3595	−0.6205
	75 °C	−1.9422	0.78998	0.184	−4.3117	0.4273

Significance level *, indicate the *p*-values < 0.05 (Tukey HSD test).
